# Investigation of the Role of Osteoporotic Vertebra Degeneration on the Stability of the Lumbar Spine: In Silico Modelling under Compressive Loading

**DOI:** 10.3390/bioengineering11050507

**Published:** 2024-05-17

**Authors:** Olga Chabarova, Jelena Selivonec, Alicia Menendez Hurtado

**Affiliations:** Department of Applied Mechanics, Vilnius Gediminas Technical University, LT-10223 Vilnius, Lithuania; olga.chabarova@vilniustech.lt (O.C.); alicia.menendez-hurtado@vilniustech.lt (A.M.H.)

**Keywords:** lumbar spine, osteoporosis, stability, intervertebral disc, FE modelling

## Abstract

An evaluation of the impact of osteoporosis on loss of spinal stability, with or without intervertebral disc degeneration, using computational analysis is presented. The research also investigates the correlation between osteoporosis and intervertebral disc degeneration. Three-dimensional finite element models of human lumbar spine segments were used to assess the influence of osteoporosis on spinal stability. Five different models of age-related degeneration were created using various material properties for trabecular bone and intervertebral discs. Calculation results indicate that in a spine with osteoporosis, the deformation of the intervertebral discs can increase by more than 30% when compared to a healthy spine. Thus, intervertebral disc deformation depends not only on the degree of degeneration of the discs themselves, but their deformation is also influenced by the degree of osteoporosis of the vertebrae. Additionally, the load-bearing capacity of the spine can decrease by up to 30% with osteoporosis, regardless of the degree of intervertebral disc deformation. In conclusion, osteoporosis can contribute to intervertebral disc degeneration.

## 1. Introduction

The spine is a flexible column consisting of a certain number of segments. Its functional role is to maintain stability and vertical position, as well as to ensure the mobility of the segments relative to each other.

When compared to other parts of the spine, the lumbar spine is the most affected by deformations, since it has to bear the majority of the weight. This part of the spine is affected not only by the maximum compression load, but also by large displacements [1,2].

Ageing affects all spinal structures. Among the diseases of the musculoskeletal system, osteoporosis, which is related to an increased risk of spinal vertebral fractures, and degenerative intervertebral disc (IVD), are pain inflicting pathologies that require large national expenses for treatment. Annually in Europe, there are 500,000 cases of broken vertebrae caused by osteoporosis [3].

Osteoporosis is a bone condition characterised by a decrease in density in the trabecular bone and bone structure degeneration [4]. The most significant decrease in bone mass caused by osteoporosis occurs in the outer layer of bone (cortical bone) through intercortical remodelling, rather than within the bone (endocortical) or in the spongy bone (trabecular bone) [5]. Trabecular bone undergoes remodelling in people under the age of 65. Over time, trabeculae become thin, horizontal bonds disappear, and gaps between the outer and inner bones appear. When trabecular bone separates from the outer shell, the amount of load on the outer shell increases, leading to a decrease in the ability of the vertebra to resist compression loads [6]. It is important to plan treatment with reliable diagnostic tools. Currently, radiographic techniques are the clinical standard for assessing noninvasive bone strength. Quantitative computed tomography (QCT) allows us to obtain the vertebra’s trimetric geometry and measure trabecular and cortical bone volumetric bone mineral density (vBMD) [7]. Although this method can evaluate bone density, it does not take into account local changes or bone morphology.

Without osteoporosis, approximately half of older people suffer from degenerative intervertebral disc disease. As the disc ages, its composition changes. The nucleus is the first part of the disc to change with age, but any change in the mechanical properties of any tissue can affect stability and load transfer mechanics [8]. Many articles concentrate on intervertebral disc research due to its central role in spinal stability [9,10,11,12,13].

As shown in a review of articles by Naoum et al. [14], many studies have used the finite element (FE) method to assess the holding capacity of osteoporotic vertebrae and the lumbar spine’s loss of stability. In articles [15,16] an osteoporotic lumbar spine FE analysis was performed, which showed a connection between the decrease in vertebral trabecular bone density and increase of spinal deformations.

Zhang [17] used the FE method to study the effect of osteoporosis on the loss of stability in the normal spine and the spine with scoliosis. Although the latest statistical studies show severe degenerative changes in the discs in people with osteoporosis [18], the relationship between intervertebral disc degeneration and osteoporosis is not sufficiently documented. Experimental analysis of vertebral degeneration is very difficult, sometimes even impossible. However, through numerical simulation, it is possible to analyse the effect of the main mechanical factors of the degeneration. In this study, the FE method was used to model osteoporosis by changing the BMD of the vertebral trabecular bone and introducing voids between the two phases, trabecular and cortical. Five models of lumbar spine segments L2–L4 were created, including a healthy lumbar spine, two lumbar spines with osteoporosis, and two lumbar spines with osteoporosis and intervertebral disc degradation. The purpose of this biomechanical study is to find a connection and potential threat between vertebrae damaged by osteoporosis and intervertebral disc deformation. This study found that, depending on the degree of damage to the trabecular bone, osteoporosis can lead to local instability of the cortical bone of the vertebrae, which in turn can lead to a loss of load-bearing capacity of the spine before reaching its ultimate strength. It has also been established that with osteoporotic changes in the vertebrae, the deformation of the intervertebral discs increases, which in turn leads to an increase in spine deformation.

## 2. Materials and Methods

### 2.1. Geometric Model

A lumbar spine section consisting of three L2–L4 vertebrae linked by intervertebral discs was tested. This fragment is the most important part of the spine, bearing a larger part of the external load which is responsible for the functionality of the human body [19]. The geometry of the model was generated using patient-specific data and the available data in references. A three-dimensional virtual model of the L2–L4 lumbar spine segment was developed in several steps. First, a 48-year-old man underwent a CT scan. The images obtained were subsequently analysed using the free open-source tool 3D Slicer (version 4.11) [20] and improved with MeshLab (version 2020.06) [21]. The resulting STL file from MeshLab [21] was transferred to the SolidWorks software platform (version 2020) [22] for final mesh rendering and conversion of surfaces into a solid model of the lumbar vertebral bodies. Posterior bone elements were added manually to accommodate hardening of the back of the vertebra. Additionally, two bony endplates were added to reflect the boundary conditions of the adjacent trabecular bone.

In a geometrically modelled vertebra, usually, the vertebral body, its cortical shell and porous core, posterior process, and the vertebra’s endplates are separated. In most cases, the vertebra body model is quasi-cylindrical, with a width of 40 mm, a depth of 33 mm, and a height of 30 mm. The thickness of the outer wall is 0.5 mm, and the thickness of the cartilage back plate is 0.5 mm [23,24]. The vertebral cortical bone, trabecular bone, endplates, and posterior bony models were modelled separately.

**Figure 1:**
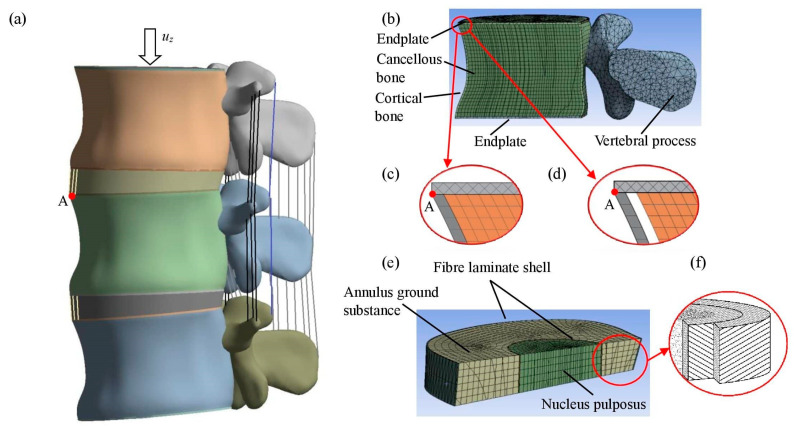
Images of the finite element (FE) models: (**a**) model of two segments (L2–L4) of the lumbar spine; (**b**) L3 vertebra; (**c**) bonded connection; (**d**) unbonded connection with a gap; (**e**) intervertebral disc; (**f**) disc layers.

The height of the geometrically modelled intervertebral disc is between 8 and 12 mm, depending on the person’s gender and height. There are three main parts in a disc model: the nucleus, the annulus ground substance, and the annulus fibres. In the lumbar spine part, the nucleus area is mostly between 30 and 50% of the total cross-sectional area of the disc [25,26]. In the aforementioned SolidWorks environment, an intervertebral disc was inserted between adjacent vertebrae, assuming a constant thickness of 10 mm along the volume. In the next step, the ligaments were incorporated into the current model components. The final numerical model of the lumbar spine segment’s L2–L4 is shown in Figure 1a.

### 2.2. Mechanical Properties

The spine is a highly heterogeneous compound structure. Because of this, modelling of the spine has to be described by giving separated parts different material properties. The material properties of the lumbar spine’s components are most often determined by specific experimental examples, and in this case, the characteristics consisted of three examples of lumbar spine material data from 54- and 69-year-old females. In this research, properties of healthy lumbar spine components were taken from CT data of the spine of a 35-year-old female.

The cortical shell is typically modelled as either linear elastic isotropic or orthotropic material. Isotropic materials have a Young’s modulus ranging from 5000 to 12,000 MPa and a Poisson’s ratio of approximately 0.2 to 0.3, while orthotropic materials have properties with a Young’s modulus of 8000 to 12,000 MPa and shear modulus of 3000 to 5000 MPa. The density of a healthy lumbar vertebra of the cortical bone is *ρ_lumbar_*_,*cor*_ = 1970 ± 200 kg/m^3^ [27]. The dense cortical shell is simulated as an orthotropic elastic-plastic continuum, with a yield stress of 64 MPa defining the limit of yielding.

Trabecular bone is described as linear elastic isotropic or orthotropic material. The modulus of trabecular bone elasticity depends on porous material density and is calculated using the formula [28]:

*E_cancellous_* = 4.730 × *ρ*^1.56^ (1)

where *ρ* (g/cm^3^) is bone density. The density of the trabecular bone is estimated with the help of the CT mean value. The trabecular bone density of a healthy vertebra is *ρ* = 300 ± 20 kg/m^3^ [29,30]. During osteoporosis, trabeculae thin out, and the horizontal bonds disappear, due to which trabecular bone density can decrease up to 50 kg/m^3^. A reduction in volume density *ρ* from 300 kg/m^3^ to 100 kg/m^3^ is a typical characteristic of osteoporotic degradation of vertebrae (Table 1).

The trabecular bone presents the largest available surface for remodelling in individuals younger than 65 years of age. When trabecular bone is removed from a vertebral body, it causes an increase in tension in critical bone and reduces the strength of the vertebra to oppose compression loads [31]. When horizontal trabeculae bonds disappear, gaps between trabecular and cortical bone appear and a bone separation effect occurs [32]. The effect of osteoporosis is modelled by inputting a space between the cortical and trabecular bones (Figure 1d).

The trabecular phase is represented as an elastic orthotropic continuum, with the assumption that the transverse elasticity modulus *E_xx_* = *E_yy_* is a fraction of the longitudinal modulus *E_zz_*, thus:

*E_xx_* = *E_yy_* = 0.1 × *E_zz_*. (2)

Spinous processes are typically modelled as linear elastic isotropic materials. The Young’s modulus of the material is between 1000 MPa and 5000 MPa with a Poisson’s ratio of *ν* = 0.2–0.25 [33,34]. Endplates are mostly described as linear elastic isotropic material, with *E* = 20–25 MPa and *ν* = 0.4 [34,35].

The material physical properties of vertebral bones are given in Table 1.

**Table 1:** Material moduli of the components.

Bone Type	Young’s Modulus [MPa]	Poisson’s Ratio
Vertebral cortical bone [36]	*E_xx_* = 2667	*ν_xy_* = 0.3
	*E_yy_* = 2667	*ν_yz_* = 0.2
	*E_zz_* = 8000	*ν_xz_* = 0.2
	*G_xy_* = 1026	
	*G_yz_* = 1539	
	*G_xz_* = 1539	
Vertebral cancellous bone	*E_xx_* = 72.3/13	*ν_xy_* = 0.3
(healthy/osteoporotic)	*E_yy_* = 72.3/13	*ν_yz_* = 0.2
	*E_zz_* = 723/130	*ν_xz_* = 0.2
	*G_xy_* = 27.8/5	
	*G_yz_* = 48.2/8.7	
	*G_xz_* = 48.2/8.7	
Vertebral bony endplate	*E* = 50	*ν* = 0.4
Posterior Bone	*E* = 3500	*ν* = 0.25

Spinal stability depends on the nucleus pulposus (NP). Its hydrostatical compression guarantees the stability of the entire disc and spine. The fluid level in the nucleus decreases with age. The pressure distribution in a degenerated disc is non-uniform and direction dependent. Usually, the material properties of NP can be described as linear elastic isotropic. The Young’s modulus of a healthy disc’s NP is about 1 MPa, with a Poisson’s ratio of *ν* = 0.499. With intervertebral disc degeneration, NP hardens and the Young’s modulus can increase to 10 MPa, with a Poisson’s ratio of *ν* = 0.49 [37].

The annulus is a common composite material made up of multiple layers of reinforced fibres in a consolidated ground substance. The material properties of the annulus ground substance include a modulus of elasticity ranging from 2 to 8 MPa and a Poisson’s ratio between 0.4 and 0.45 [34,38]. A more recent model takes material nonlinearities with a Neo-Hookean formulation for the annulus matrix [39,40]. The model is assigned a Neo-Hookean material relationship, and the values of *C*_10_ and *D* are adjusted within a specified range [41]:

(3) $$C_{10}=\frac{\mu_{0}}{2},$$

(4) $$D=\frac{2}{K_{0}},$$

where

(5) $$\mu_{0}=\frac{E}{2\times \left(1+\upsilon \right)},$$

(6) $$K_{0}=\frac{E}{3\times \left(1+2\times \upsilon \right)}.$$

Disc degradation is characterised by an elasticity modulus *E* between 1.4 MPa and 6.3 MPa, with a Poisson’s ratio of 0.4.

All major ligaments (Anterior Longitudinal (ALL), Posterior Longitudinal (PLL), Capsular (CL), Ligamentum Flavum (LF), Interspinous (ISS), and Supraspinous (SSL)) were designed. To construct the ligaments, three-dimensional two-node link elements were used. The mechanical properties were taken from [37]. Young’s modulus values of the ligaments are $E_{ALL}=20$ MPa, $E_{PLL}=20$ MPa, $E_{CL}=33$ MPa, $E_{LF}=19$ MPa, $E_{ISS}=12$ MPa, $E_{SSL}=12$ MPa. Poisson’s ratio of the ligaments is $\nu_{L}=0.3$.

Five grades of data from the normal ageing degeneration process are shown in Table 2, ranging from a healthy case to a degenerated case.

### 2.3. FE Model

An experiment was carried out using the finite element method computer program ANSYS (version 19.1) Newton-Raphson Method to perform calculations.

The equilibrium of complex structures is formulated with energy statements instead of differential equations. Specifically, the principle of virtual displacements is used. The incremental formulation of this model is defined at time instant *t* as follows:

[**K***e* + **K***σ* + **K***u* + **K***nl*1 + **K***nl*2] Δ**u** = Δ**F**, (7)

where **K***_e_* is the elastic element stiffness matrix; **K***_σ_* is the geometric stiffness matrix; **K***_u_* is the initial displacement matrix; **K***_nl_*_1_, **K***_nl_*_2_ are large displacement matrices that depend on Δ**u**; Δ**u** and Δ**F** are increments of displacement and external load vectors.

The model includes cortical and trabecular vertebral bones, as well as endplates, spinous processes, the nucleus and annulus of the intervertebral disc (Figure 1a). The body was modelled as porous bone continuum surrounded by cortical bone shell. The models of trabecular bone, endplate, and spinous process were meshed using volumetric finite elements (Figure 1b).

A hierarchical multilevel FEM design strategy is proposed for assessing the impact of vertebral pathological changes on the stability of the spine and its elements. To understand the general biomechanics of the spine, it is essential to assess the mechanical behaviour of individual vertebrae. For that purpose, the FE model of the vertebra of the lumbar spine is considered. The FE model was generated as described below (Figure 1c). When studying the model, a mesh with an element size of 2 mm was used. The cortical bone of the vertebra was discretised by shell FE. Models of vertebral trabecular bone, cartilage plates, and processes were segmented by volumetric FE. These types of solids are higher-order 3D 20-node solids with second-order displacement behaviour. The element is ductile, capable of large displacements and large deformations. The cortical shell and the trabecular bone are connected by nodes connecting the translational degrees of freedom. The cortical bone’s thin-walled domain was divided into shell finite elements. The FE mesh of the cortical shell contains 11,665 shell elements with 11,882 nodes. The patterns of cancellous bone, cartilage plates, and vertebral outgrowths were divided by volumetric FE. The solid phase was finally represented by a 3D mesh consisting of 147,814 solid elements and 348,138 nodes.

For the analysis of the global stability of the spine, the FE model of two movable segments of the lumbar spine is considered (Figure 1a). The model consists of dense and spongy vertebral bones, cartilaginous plates, bony processes of the vertebra, intervertebral disc annulus, nucleus, and annulus fibres. The intervertebral disc consists of the annulus ground substance, the annulus fibrosus, and the nucleus pulposus. In this study, the fibre annulus has two layers of fibre bundles that are arranged in +30° and −30° layers. Composite four-node shell elements were used in this study to model annular fibres and assess their resistance to in-plane forces. The thickness of the fibre shell reaches 1.5 mm, with the focus solely on in-plane behaviour while disregarding bending stiffness. The FE model is presented (Figure 1f). The FE mesh of cortical shell and ring fibres contains 9841 shell elements with 10,028 nodes. Vertebral trabecular bone, cartilaginous plates, processes, intervertebral disc annulus ground substance and nucleus were segmented by 3D finite elements. The model contains 188,100 volume elements with 710,751 nodes. Each shell element has four nodes with six degrees of freedom at each node: linear displacements in the x, y, and z directions, and angular displacements about the x, y, and z axes. Elements can be connected through nodes, using both centreline and outer nodes. Such elements are non-planar, associated with plasticity and large deformation, and describe the curvature of the structure. They are suitable for analysing thin to medium-thickness shell structures. The ligaments are represented as tensile-only uniaxial link elements. Each ligament element has two nodes with three degrees of freedom at each node: linear displacements in the x, y, and z directions. Tension-only options are supported. Creep, plasticity, rotation, large strain, and large deflection capabilities are included. Link elements allow for a change in cross-sectional area as a function of axial elongation (Figure 1a).

The connection between the cortical wall and trabecular phase was implemented as a contact between two solids (Figure 1c), which is modelled as a bonded contact in the perfect case, with no sliding or separation between faces or edges. In the case of the shell-solid constraint, force-distributed constraints are created between nodes on the solid surface and on the shell edges. These nodes on the shell edges act as master nodes, while associated solid nodes act as slave nodes.

The bond is weakened in the osteoporotic degradation case, and it may disappear in the limit case. The degradation effect could be evaluated by removing the connecting bonds (Figure 1d).

Kinematic boundary conditions control the behaviour of the finite element model. At the bottom of L4, movement is zero, and the load increases proportionally up to the upper endplate of L2, which has a maximum vertical displacement of *u_z_*(*t*). Therefore, the contribution of instantaneous displacement controls the external axial load at a given time *t*. This load is controlled by the monotonically increasing displacement of the upper endplate *u_z_*(*t_max_*) = *u_z,max_*, which is limited by the maximum value of *u_z,max_* = 2.5 mm. The load is transferred to the trabecular and cortical bones via an endplate.

## 3. Results

Each of the five models was analysed under the influence of purely axial loading. The time interval was set as 0 ≤ $\bar{t}$ *≤* 1, and the specified displacement of the upper endplate controlled the axial loading.

### 3.1. Failure Mechanisms of the Two Lumbar Segments

Table 2 details the simulation of degeneration of the lumbar spine associated with aging. Osteoporotic vertebral deterioration is identified by a reduction in porous bone density, along with the separation of cortical and trabecular bone.

A healthy intravertebral disc nucleus is in hydrostatic compression state. With age, it loses its resistance to compression. Decreasing Poisson’s ratio as Young’s modulus increases can simulate nucleus degeneration [42].

Statistical data and experiments show that fractures occur most often in vertebrae affected by osteoporosis [43]. Spine strength (*F*_max_) was taken when the maximum load was reached, compressing the lumbar spine 2.5 mm.

**Figure 2:**
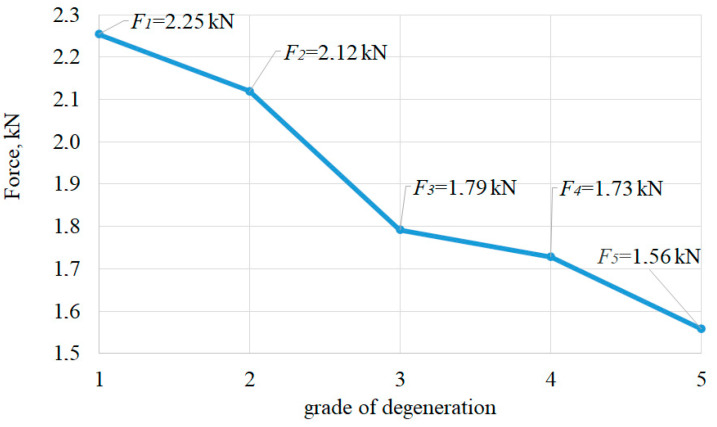
Maximum lumbar segment forces for 2.5 mm compression versus age-related degeneration.

In Figure 2 maximal compression force alteration is shown, which is dependent on the degree of lumbar spine degeneration, in which lumbar spine is compressed 2.5 mm. When the trabecular bone is removed from a vertebral body, it results in heightened stresses on the cortical shell and a greater reduction in the bone’s ability to resist compression forces [31], corresponding to the experimental data [44].

**Figure 3:**
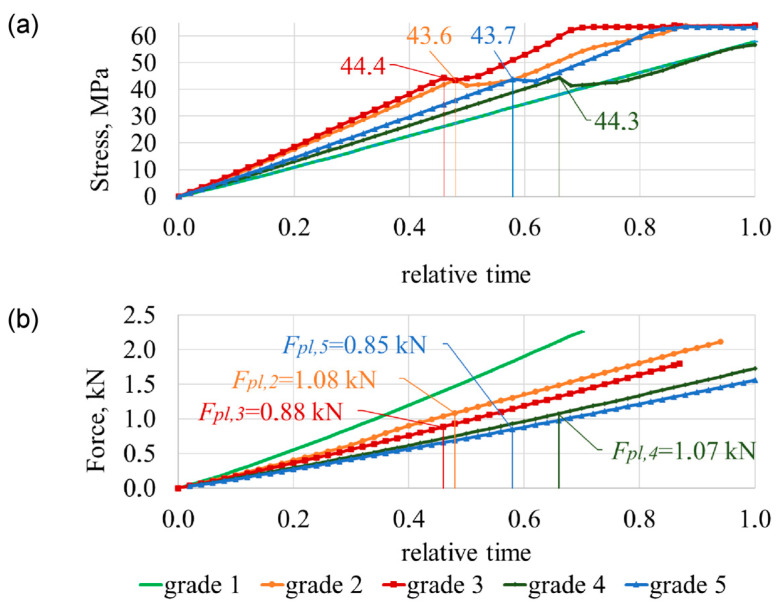
Variation in time of 2.5 mm vertical load (**a**) of cortical shell von Mises tension and (**b**) of compression force.

Figure 3 shows the comparison of different modules by charting the time histories of the selected displacement and force parameters during the entire loading period for $0\leq \bar{t}\leq 1$. Here, the variation of compression load is plotted against relative time $\bar{t}=t/t_{max}$. The relative time ranges between the 0 and 1 interval ($0<\bar{t}<1$) and illustrates the behaviour of the structure during the loading state. By solving Equation (7), the stress-strain state of the body is obtained, given the loads and boundary conditions. For the purpose of illustrating the results, a selection of stresses, strains, and displacements may be explored. In the case of plastic deformation, the load carrying capacity of the material increases as the deformation increases due to strain hardening. Variations of the von Mises of the cortical shell are illustrated in Figure 3a and of the maximum carrying load at which plastic deformations begin are illustrated in Figure 3b.

**Figure 4:**
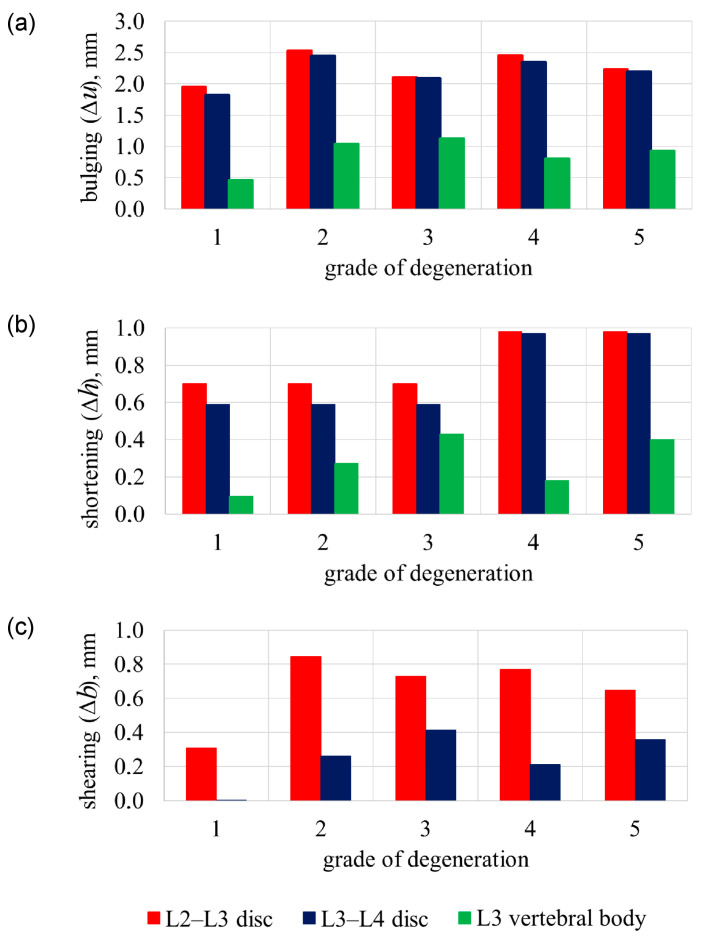
Effect of age-related degeneration of 2.5 mm vertical load: (**a**) discs and cortical shell bulging; (**b**) discs and vertebra shortening; (**c**) discs shearing.

Figure 4a shows the disc and vertebral cortical bone bulging and Figure 4b the disc and vertebral body shortening in terms of ageing degeneration.

With numerical results, while compressing a healthy lumbar spine (grade 1), the compression force was *F*_1_ = 2.25 kN (Figure 2). The tensions of the cortical shell are distributed according to linear dependency (Figure 3a). Cortical shell bulging is fairly small Δ*u*_L3,1_ = 0.46 mm. Consequently, L2–L3 disc bulging lateral direction is Δ*u*_L2–L3,1_ = 1.81 mm, L3–L4 disc bulging is Δ*u*_L3–L4,1_ = 1.95 mm (Figure 4a), discs shearing is Δ*b*_L2–L3,1_ = 0.001 mm and Δ*b*_L3–L4,1_ = 0.31 mm (Figure 4c). When the 2.5 mm compression of the lumbar segment occurred, the height decreased only by Δ*h*_L3,1_ = 0.095 mm, and the height of the intervertebral discs decreased by Δ*h*_L2–L3,1_ = 0.59 mm and Δ*h*_L3–L4,1_ = 0.7 mm (Figure 4b).

The focus shifts to displacement-based criteria when considering osteoporotic degeneration of the lumbar spine. When compressing the osteoporotic lumbar (grade 2), with a healthy disc, the compression force decreased slightly and is *F*_2_ = 2.12 kN (Figure 2). In the cortical shell during the relative time’s moment ${\bar{t}}_{pl,2}=0.6$, affected by force *F_pl_*_,2_ = 1.08 kN, plastic deformations appear when strength capacity was not reached *σ_pl_*_,2_ = 43.6 MPa < 64 MPa (Figure 3). Although the compression force changed slightly when compared with a healthy vertebra, cortical shell bulging increased more than two times up to Δ*u*_L3,2_ = 1.04 mm, i.e., 126% more than a healthy vertebra. Additionally, L2–L3 disc’s bulging increased 35%, i.e., Δ*u*_L2–L3,2_ = 2.45 mm, and L3–L4 disc’s bulging increased 30%, i.e., Δ*u*_L3–L4,2_ = 2.53 mm (Figure 4a). L2–L3 disc’s shearing increased Δ*b*_L2–L3,2_ = 0.26 mm, i.e., 260% more than grade 1, and L3–L4 disc’s shearing increased Δ*b*_L3–L4,2_ = 0.85 mm, i.e., 174% more than grade 1 (Figure 4c). The difference in disc height between a compressed grade 2 lumbar segment, and a healthy disc does not differ from grade 1: Δ*h*_L2–L3,2_ = 0.59 mm and Δ*h*_L3–L4,2_ = 0.7 mm. In this case, osteoporotic vertebra’s height changed Δ*h*_L3,2_ = 0.27 mm, i.e., 185% more than a healthy vertebra (Figure 4b).

With gaps between the trabecular and cortical bones and with a healthy disc (grade 3), the compression force decreased by 26%, up to *F*_3_ = 1.79 kN (Figure 2). In relative time’s moment ${\bar{t}}_{pl,3}=0.6$, with compression force *F_pl_*_,3_ = 0.88 kN and *σ_pl_*_,3_ = 44.4 MPa < 64 MPa, cortical shell displays plastic deformations (Figure 3). Although discs height difference Δ*h*_L2–L3,3_ = Δ*h*_L2–L3,1_ = 0.59 mm and Δ*h*_L3–L4,3_ = Δ*h*_L3–L4,1_ = 0.7 mm, unbonded vertebra’s height decreased Δ*h*_L3,3_ = 0.43 mm, i.e., 350% more than a healthy vertebra’s (Figure 4b). L2–L3 disc’s shearing increased Δ*b*_L2–L3,3_ = 0.41 mm, i.e., 410% more than a healthy spine segment and L3–L4 disc’s shearing increased Δ*b*_L3–L4,3_ = 0.73 mm, i.e., 109% more than a healthy spine segment. With a compressed grade 3 lumbar, the bulging of the vertebral wall is Δ*u*_L3,3_ = 1.12 mm, i.e., 146% more than a healthy vertebra. Disc’s bulging (grade 3) increases up to Δ*u*_L2–L3,3_ ≈ Δ*u*_L3–L4,3_ = 2.1 mm (Figure 4a).

When disc degeneration occurs and vertebrae are affected by osteoporosis (grade 4), to compress the lumbar 2.5 mm, a force of *F*_4_ = 1.74 kN is required, which is 30% smaller, than when compared with grade 1 (Figure 2). When reached *F_pl_*_,4_ = 1.07 kN (Figure 3b), during relative time’s moment ${\bar{t}}_{pl,4}=0.84$, vertebra starts to show plastic deformations with tensions smaller than the strength’s capacity *σ_pl_*_,4_ = 44.3 MPa < 64 MPa (Figure 3a). Vertebra’s cortical bone bulging is Δ*u*_L3,4_ = 0.8 mm, i.e., 74% more than grade 1. L2–L3 disc’s bulging increased by 30%, when compared with grade 1, and is Δ*u*_L2–L3,4_ = 2.34 mm, and L3–L4 disc’s bulging increased by 27%—Δ*u*_L3–L4,4_ = 2.45 mm. Additionally, L2–L3 disc’s shearing increased by 210%, when compared with grade 1, and is Δ*b*_L2–L3,4_ = 0.21 mm, and L3–L4 disc’s shearing increased by 148%—Δ*b*_L3–L4,4_ = 0.77 mm (Figure 4c). The height difference is similar to that of grade 2 and is Δ*h*_L3,4_ = 0.18 mm. Additionally, L2–L3 degenerated disc height difference changed by 64% and is Δ*h*_L2–L3,4_ = 0.97 mm, and L3–L4 disc height difference is Δ*h*_L3–L4,4_ = 0.98 mm, i.e., 40% more than a healthy spine (Figure 4b).

The adverse case is when there is osteoporotic vertebral cortical and trabecular bone separation, with a degenerated disc (grade 5). To compress such lumbar 2.5 mm, a force 44% smaller when compared with healthy spine is required. This force is only *F*_5_ = 1.56 kN (Figure 2). With force *F_pl_*_,5_ = 0.85 kN, during relative time’s moment ${\bar{t}}_{pl,5}=0.76$ (Figure 3b), vertebral wall shows plastic deformations with *σ_pl_*_,5_ = 45.4 MPa < 64 MPa (Figure 3a). Vertebral wall’s bulging is Δ*u*_L3,5_ = 0.92 mm, and discs accordingly are Δ*u*_L2–L3,5_ = 2.20 mm, Δ*u*_L3–L4,5_ = 2.23 mm. Vertebra’s grade 5 height difference is similar to grade 3, Δ*h*_L3,5_ = 0.4 mm, and grade 5 degenerated discs height difference are same as grade 4, i.e., Δ*h*_L2–L3,5_ = Δ*h*_L2–L3,4_ = 0.98 and Δ*h*_L3–L4,5_ = Δ*h*_L3–L4,4_ = 0.97 mm (Figure 4b). L2–L3 disc’s shearing is Δ*b*_L2–L3,5_ = 0.36 mm, i.e., 360% more than grade 1, and L3–L4 disc’s shearing is Δ*b*_L3–L4,5_ = 0.65 mm, i.e., 110% more than grade 1 (Figure 4c).

### 3.2. L3 Vertebra’s Failure Mechanisms

**Figure 5:**
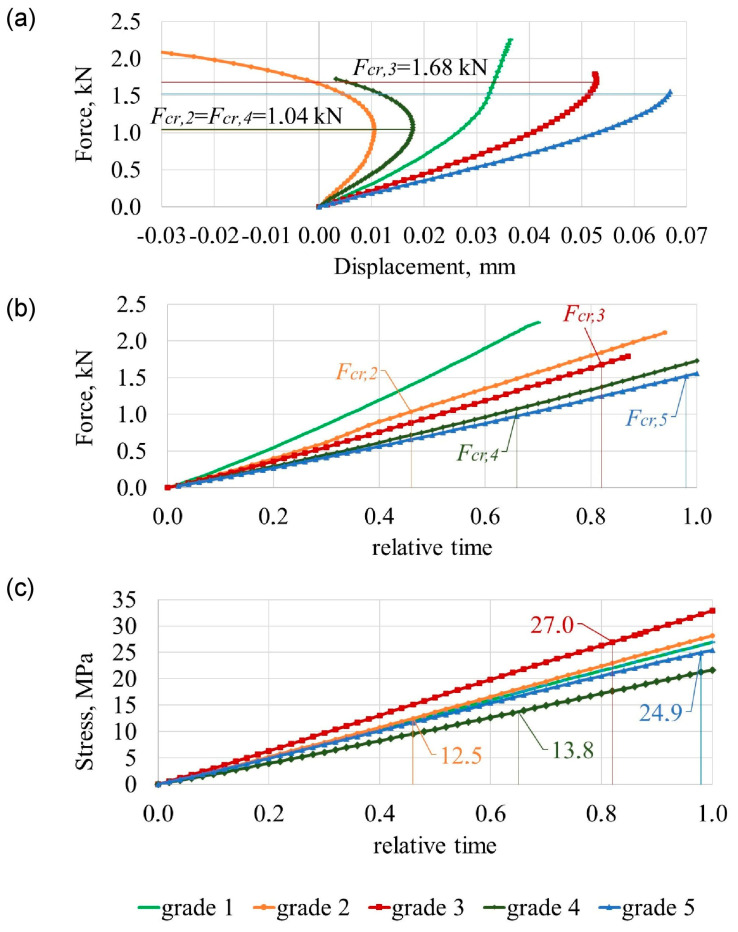
Changes at point A: (**a**) of forces-horizontal displacement; (**b**) of compression force in time; (**c**) of von Mises tension in the cortical shell in time.

The loads at which vertebral buckling begins are considered critical. At this load, critical displacements occur at which the thin cortical bone of the vertebrae undergoes a significant change in behaviour, resulting in buckling, which may lead to vertebral structural instability. The presence of critical loads was confirmed by considering the variation of horizontal displacement *u_x_*(*t*) at point A (Figure 5a). It was found that the presence of a perfect bond shows a different behaviour of the critical loads. Thin lines illustrate the first bifurcation point, which indicates critical displacement on a horizontal axis and critical load on a vertical axis.

Essential properties of the compressed body are characterised by the force–displacement relationship. The numerical results are shown in Figure 5b. The relative time scale also reflects displacement (0 < *u*(*t*) *< u_z_*_,max_(*t*_max_)).

The curves denoted as grade 2, grade 4 illustrate the variation of specified quantities corresponding in the case of perfect bonding. The next curves, grade 3, grade 5, analogously illustrate the time variation of these quantities for the case of a degenerated bond considering displacement (Figure 5) of the critical loads *F_cr_*_,2_, *F_cr_*_,3_, *F_cr_*_,4_, *F_cr_*_,5_.

On the basis of numerical results (grade 2, grade 4) obtained for unbonded vertebrae under axial load, it was found that the strength criterion is what characterises the load-bearing capacity of the vertebrae. For the case of purely axial compression, the time history of the von Mises stress *σ* in Figure 3a shows that the strength criterion of *σ_y_* ≈ 64 MPa is satisfied at relative time instant ${\bar{t}}_{cr,3}=0.82$, ${\bar{t}}_{cr,5}=0.98$.

Therewith, local displacements $u_{x,3}\left({\bar{t}}_{cr,3}\right)=0.05$ mm, $u_{x,5}\left({\bar{t}}_{cr,5}\right)=0.07$ mm (Figure 5a) are relatively big, while the load at time instant ${\bar{t}}_{cr,3}$, ${\bar{t}}_{cr,5}$ may be considered as limit load *F_cr_*_,3_ = 1.68 kN and *F_cr_*_,5_ = 1.52 kN (Figure 5b). The distribution of von Mises stress (Figure 3a) clearly confirms this statement.

The presence of critical loads $F_{cr,2}\left({\bar{t}}_{cr,2}=0.46\right)=1.04$ kN < $F_{pl,2}\left({\bar{t}}_{pl,2}=0.48\right)=1.08$ kN, $F_{cr,3}\left({\bar{t}}_{cr,3}=0.82\right)=1.68$ kN > $F_{pl,3}\left({\bar{t}}_{pl,3}=0.46\right)=0.88$ kN, $F_{cr,4}\left({\bar{t}}_{cr,4}=0.65\right)=1.04$ kN < $F_{pl,4}\left({\bar{t}}_{pl,4}=0.66\right)=1.07$ kN, $F_{cr,5}\left({\bar{t}}_{cr,5}=0.98\right)=1.52$ kN > $F_{pl,5}\left({\bar{t}}_{pl,5}=0.58\right)=0.85$ kN was confirmed by considering the variation of horizontal displacement *u_x_*(*t*) at point A (Figure 5a). These loads are characterised by elastic state, $\sigma \left({\bar{t}}_{cr,2}\right)=41.8$ MPa < 64 MPa, $\sigma \left({\bar{t}}_{cr,3}\right)=63.3$ MPa ≈ 64 MPa, $\sigma \left({\bar{t}}_{cr,4}\right)=43.6$ MPa < 64 MPa, $\sigma \left({\bar{t}}_{cr,5}\right)=63.2$ MPa ≈ 64 MPa (Figure 3b).

Bifurcation points stand for total stability load with unstable post-buckling behaviour. The change in horizontal displacement at point A, represented by *u_x_*(*t*), is responsible for the post-buckling behaviour following bifurcation. The decrease in horizontal displacement shown in Figure 5a indicates unstable motion after buckling. The global instability post-buckling is characterised by a unlimited decrease in displacement (categories 2, 3, 4, 5).

## 4. Discussion

Currently, lumbar instability is considered a significant public health issue that not only increases the morbidity of older adults, but also brings about important economic costs [45]. The appearance of instability in the lumbar region leads to increased movement between the vertebrae and initiates the progression of the degeneration of the intervertebral discs. Instability in the degenerative lumbar intervertebral region plays a crucial role in determining when spinal surgery is necessary [46].

**Figure 6:**
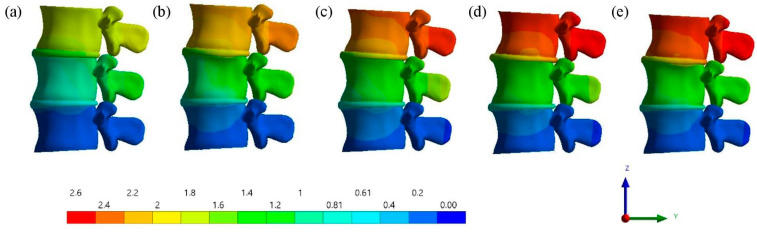
Deformed shapes of the L3 cortical shell in a frontal view, along with a contour plot of the total deformation (mm) after loading at time instant $\bar{t}$ = 1: (**a**) grade 1; (**b**) grade 2; (**c**) grade 3; (**d**) grade 4; (**e**) grade 5.

This study showed that lumbar vertebrae with different degrees of osteoporosis exhibit different displacements. Furthermore, the comparison of the contour plot between the deformed shapes of the L2–L4 lumbar spine segments (as shown in Figure 6) emphasize that the displacement of the lumbar vertebrae with osteoporosis is greater than that of the lumbar vertebrae without osteoporosis. With a vertical load of 2.5 mm, the displacement of healthy vertebrae is 1.8 mm, and the displacement of osteoporotic vertebrae increases by 30% to 2.34 mm. Local damage to the trabecular bone further increases this process and the displacement of the lumbar vertebrae becomes 2.52 mm, which is accordingly 40% greater than that of healthy lumbar vertebrae. The results showed that in osteoporotic lumbar vertebrae with intervertebral disc degeneration, the rate of increase in patient displacement was significantly higher than in osteoporotic lumbar vertebrae without intervertebral disc degeneration. The mixing increased by 40% to 2.61 mm. This can be explained by the deformation of the vertebrae, as a result of which, additional shear forces begin to act on the intervertebral disc, causing its degeneration [47]. Therefore, an increase in spinal deformity due to osteoporosis can lead to spinal instability [48]. Physiotherapists are able to detect displacements in the posterior process of the vertebra, as supported by the findings of an in vivo study [49].

**Figure 7:**
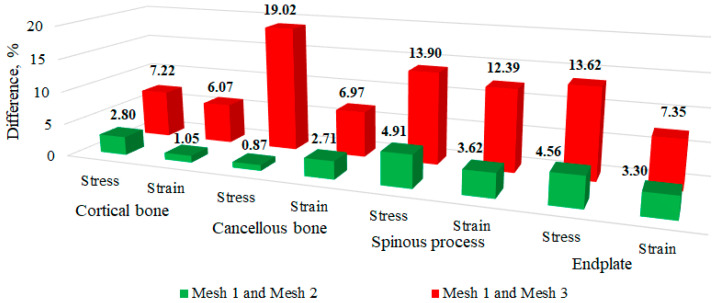
The percentage variations in von Mises stresses and strains observed in various tissues when comparing Mesh 1 with Mesh 2 and Mesh 1 with Mesh 3 under compression loading.

We validated our finite element model by comparing stresses and strains in individual vertebral elements with experimental data [50]. The mesh convergence of one of the five FE models was analysed, with a three-dimensional model of an osteoporotic vertebra developed to accurately depict the internal geometry of the vertebral body. The model reflected osteoporotic degradation by incorporating a trabecular bone density of 0.45 g/m^2^ [50]. The 3D geometric model was imported into ANSYS 19.1 software. Three different mesh resolutions (1 mm, 2 mm, and 3 mm) were tested under the same compression load. A mesh was considered convergent if the results from two successive mesh resolutions varied by no more than 5% [34]. Figure 7 illustrates the percentage variances in the strain and the von Mises stress between Mesh 1 and Mesh 2, as well as between Mesh 1 and Mesh 3. The differences in the von Mises stresses and strains between Mesh 1 and Mesh 2 were found to be below 5% for all the vertebral elements of the model. Consequently, Mesh 2 is deemed to be a reliable approximation for stress and strain (see Figure 7). The resultant deformation of the model, utilizing a mesh size of 2 mm, was 5.10%, which is slightly lower than the experimental data of 5.14% reported by Kurutz et al. [50].

**Figure 8:**
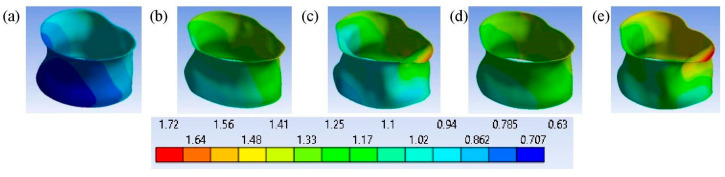
The deformed shapes of the L3 cortical shell in a front view, along with a contour plot of the total deformation (mm) after loading at time instant $\bar{t}$ = 1: (**a**) grade 1; (**b**) grade 2; (**c**) grade 3; (**d**) grade 4; (**e**) grade 5.

**Figure 9:**
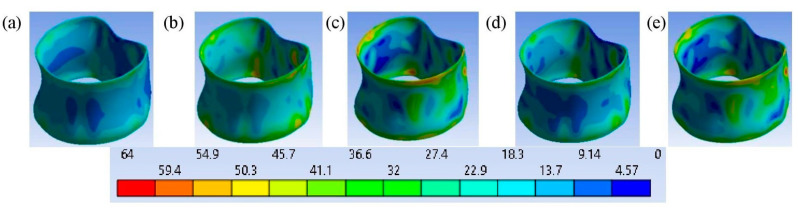
The von Mises stress σ (MPa) distribution on the L3 cortical shell after loading at time instant $\bar{t}$ = 1: (**a**) grade 1; (**b**) grade 2; (**c**) grade 3; (**d**) grade 4; (**e**) grade 5.

Upon the application of a 2.5 mm displacement, the distribution of the von Mises stress (Figure 8) and deformation of the cortical shell (Figure 9) demonstrates that the maximum values of deformation of the cortical bones of the vertebrae are concentrated within grade 3 and grade 5, leading to a concentration of stresses in the cortical layer of the bone due to increased deformation. In this way, changes in the structure or properties of bone material, or the inability to adapt these structural and mechanical properties of materials to loads, lead to bone deformation [51].

**Figure 10:**
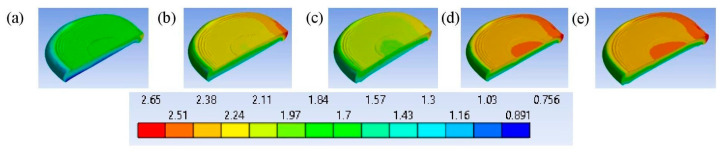
The deformed shapes of the intervertebral disc in a front view, along with a contour plot of the total deformation (mm) after loading at time instant $\bar{t}$ = 1: (**a**) grade 1; (**b**) grade; (**c**) grade; (**d**) grade 4; (**e**) grade 5.

Figure 10 shows how the deformed shapes of the intervertebral discs represent the physical nature of various models. Figure 10 shows that the deformation of the intervertebral discs depends not only on their degree of degeneration but is also influenced by the degree of osteoporosis of the vertebrae [47]. It also shows that with the same axial deformation of the entire model, at grade 3, the deformation of the vertebra increased, and as a result, less deformation was transferred to the intervertebral disc compared to at grade 2.

Our study, as well as the study by other scientists [17], showed that with osteoporosis, the risk of lumbar spine instability increases.

The compressive strength of the osteoporotic lumbar spine varies between 0.9 kN and 4.3 kN, depending on sex, age, and body mass [50,52]. According to our research, individuals with osteoporosis can have a decrease in load bearing capacity of the spine of up to 20%. Degradation of the intervertebral discs further affects loss of load bearing capacity of the spine, which can decrease by up to 30%.

The presence of local degradation between cortical and trabecular bone was found to have catastrophic consequences for the mechanical behaviour of vertebrae by performing numerical stability analyses of the lumbar spine. The local instability caused by buckling is a result of the disappearance of bonds. With trabecular and cortical bone separation, the vertebra also exhibits a buckling effect when subjected to a load of only 1.04 kN. This is two times less than the force obtained by compressing a healthy spine without osteoporosis.

With deformation of osteoporotic vertebrae, intervertebral disc deformation can increase in lateral direction more than 30%.

The importance of osteoporotic vertebrae buckling in loss of stability in the lumbar spine has been confirmed, which can aid in the development of effective treatment strategies. To fully utilise the model and proposed method in clinical practice, more research on biological samples is necessary to compare the mechanical properties of spinal components with degenerative processes.

There were a few constraints in our lumbar L2–L4 finite element model. Firstly, we did not confirm the accuracy of our FEM technique through in vitro mechanical experiments, opting instead to rely on data from the existing literature [50]. Secondly, we did not incorporate muscle strength into our model as we concentrated solely on static compression loads, neglecting dynamic forces that may impact osteoporosis-induced lumbar damage. Thus, more investigation is needed to understand the implications on the surrounding vertebrae and the overall spinal column.

As the spine ages, the vertebral bodies may become deformed due to degeneration, causing a distortion in the spine’s configuration. Fractures of the endplate or vertebral body can further contribute to this deformation. Degenerative changes in the discs, ligaments, and muscles can result in an imbalance and abnormal movement. Ultimately, these changes can lead to spinal instability, either in specific segments or throughout the spine. This instability can progress to the development of deformities such as pronounced kyphosis, degenerative scoliosis, decreased lordosis, or a combination of these issues [53].

In conclusion, the changes in the spine that occur due to aging and various factors can lead to pain and disability, although they may not always be symptomatic. It can be challenging for physicians to determine if a patient’s symptoms are directly related to radiologic findings or are more general. This difficulty is amplified when multiple levels are affected. In orthopedic practice, alongside physical examination, the consideration of radiological data can play a crucial role in establishing an accurate diagnosis, as it allows for the evaluation of both changes in the mechanical properties of materials and changes in geometric parameters.

## Figures and Tables

**Table 2:** Analysis of material moduli of components in segments as they progress from healthy (1) to degenerated (5) phases provides a model for age-related degeneration processes.

Grades of Age-Related Degeneration *	Grade 1	Grade 2	Grade 3	Grade 4	Grade 5
Cancellous bone (density [kg/m^3^])	300	100	100	100	100
Cortical bone	bonded	bonded	unbonded	bonded	unbonded
Nucleus (Elastic Modulus [MPa]/Poisson’s Ratio, *ν*)	1/0.4999	1/0.4999	1/0.4999	1.66/0.4	1.66/0.4
Annulus ground substance (Coefficients of Neo-Hookean material/Poisson’s Ratio, *ν*)	*C*_10_ = 0.25; *D*_1_ = 0.86/0.40	*C*_10_ = 0.25; *D*_1_ = 0.86/0.40	*C*_10_ = 0.25; *D*_1_ = 0.86/0.40	*C*_10_ = 1.13; *D*_1_ = 0.19/0.40	*C*_10_ = 1.13; *D*_1_ = 0.19/0.40
Annular Fibres (external/internal)	500/300	500/300	500/300	500/300	500/300

* Bony elements are shown in Table 1.

## Data Availability

The data presented in this study are available on request from the corresponding author.
